# 1,4-Bis[(5-phenyl-1,3,4-thia­diazol-2-yl)sulfan­yl]butane

**DOI:** 10.1107/S1600536811006064

**Published:** 2011-02-23

**Authors:** Shao-feng Li, Jing-jing Zhang, Xiao-yu Jia, Yan Gao, Wei Wang

**Affiliations:** a School of Chemical Engineering, University of Science and Technology LiaoNing, Anshan 114051, People’s Republic of China; b School of Perfume and Aroma Technology, Shanghai Institute of Technology, Shanghai 200235, People’s Republic of China

## Abstract

The asymmetric unit of the title compound, C_20_H_18_N_4_S_4_, contains one half-mol­ecule situated on a twofold rotation axis, in which the thia­diazole and phenyl rings are twisted by 7.2 (3)°. In the crystal, weak inter­molecular C—H⋯π inter­actions link the mol­ecules into layers parallel to (103).

## Related literature

For the biological activity of 1,3,4-triazole derivatives, see: Nakagawa *et al.* (1996 ▶); Wang *et al.* (1999 ▶). For the crystal structure of bis­(5-phenyl-1,3,4-thia­diazol-2-ylsulfan­yl)meth­ane, see: Wang *et al.* (2010 ▶).
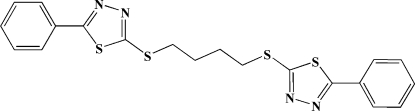


## Experimental

### 

#### Crystal data

C_20_H_18_N_4_S_4_
*M* *_r_* = 442.62
Monoclinic, 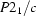

*a* = 5.7976 (7) Å
*b* = 13.4393 (14) Å
*c* = 12.9784 (12) Å
β = 99.120 (7)°
*V* = 998.44 (18) Å^3^
*Z* = 2
Mo *K*α radiation
μ = 0.49 mm^−1^
*T* = 113 K
0.20 × 0.18 × 0.10 mm

#### Data collection

Rigaku Saturn CCD area-detector diffractometer
Absorption correction: multi-scan (*CrystalClear*; Rigaku/MSC, 2005 ▶) *T*_min_ = 0.908, *T*_max_ = 0.953
9992 measured reflections
2384 independent reflections
1870 reflections with *I* > 2σ(*I*)
*R*_int_ = 0.038

#### Refinement

*R*[*F* ^2^ > 2σ(*F* ^2^)] = 0.031
*wR*(*F* ^2^) = 0.088
*S* = 1.06
2384 reflections
127 parameters
H-atom parameters constrained
Δρ_max_ = 0.49 e Å^−3^
Δρ_min_ = −0.22 e Å^−3^

### 

Data collection: *CrystalClear* (Rigaku/MSC, 2005 ▶); cell refinement: *CrystalClear*; data reduction: *CrystalClear*; program(s) used to solve structure: *SHELXS97* (Sheldrick, 2008 ▶); program(s) used to refine structure: *SHELXL97* (Sheldrick, 2008 ▶); molecular graphics: *SHELXTL* (Sheldrick, 2008 ▶); software used to prepare material for publication: *SHELXTL*.

## Supplementary Material

Crystal structure: contains datablocks global, I. DOI: 10.1107/S1600536811006064/cv5053sup1.cif

Structure factors: contains datablocks I. DOI: 10.1107/S1600536811006064/cv5053Isup2.hkl

Additional supplementary materials:  crystallographic information; 3D view; checkCIF report

## Figures and Tables

**Table 1:** Hydrogen-bond geometry (Å, °) *Cg* is the centroid of the C1–C6 ring.

*D*—H⋯*A*	*D*—H	H⋯*A*	*D*⋯*A*	*D*—H⋯*A*
C9—H9*B*⋯*Cg*^i^	0.99	2.70	3.540 (2)	144

Symmetry code: (i) 
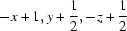
.

**Fig. 1.**
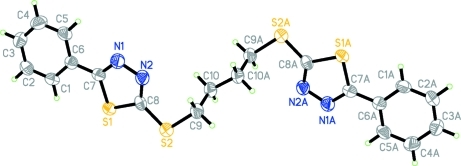
View of the molecule of (I) showing the atom-labelling scheme [symmetry code: (A)-x + 1, -y + 1, -z]. Displacement ellipsoids are drawn at the 85% probability level.

## supplementary crystallographic information

### Comment

1,3,4-Thiadiazole derivatives exhibit a wide spectrum of biological activities (Nakagawa *et al.*, 1996; Wang *et al.*, 1999). Recently, we have published the crystal structure of bis(5-phenyl-1,3,4-thiadiazol-2-ylsulfanyl)methane (Wang *et al.*, 2010). Herewith we report the crystal structure of the title compound, (I).

In (I) (Fig. 1), the molecule is situated on a twofold rotational axis so asymmetric unit contains a half of the molecule. The dihedral angle between the thiadiazole and the attached benzene rings is 7.2 (3)° indicating that two rings are almost parallel. As a result of π-π conjugation, the C*_sp_*^2^-S bond [S2—C8 = 1.742 (2) Å] is significantly shorter than the C*_sp_*^3^-S bond [S2—C9 = 1.813 (2) Å].

In the crystal structure, weak intermolecular C—H···π interactions (Table 1) link molecules into layers parallel to (103) plane.

### Experimental

A suspension of 5-diphenyl-1,3,4-thiadiazol-2-thiol (2.0 mmol) and 1,1-dibromobutane (1.0 mmol) in ethanol (10 ml) was stirred at room temperature. The reaction progress was monitored *via* TLC. The resulting precipitate was filtered off, washed with cold ethanol, dried and purified to give the target product as light yellow solid in 85% yield. Crystals of (I) suitable for single-crystal X-ray analysis were grown by slow evaporation of a solution in chloroform-ethanol (1:1).

### Refinement

All H atoms were positioned geometrically (C—H = 0.95–0.99 Å) and refined as riding, with *U*_iso_(H) = 1.2*U*_eq_(C).

### Crystal data

**Table:**

C_20_H_18_N_4_S_4_	*F*(000) = 460
*M**_r_* = 442.62	*D*_x_ = 1.472 Mg m^−^^3^
Monoclinic, *P*2_1_/*c*	Mo *K*α radiation, λ = 0.71073 Å
Hall symbol: -P 2ybc	Cell parameters from 3630 reflections
*a* = 5.7976 (7) Å	θ = 1.5–27.9°
*b* = 13.4393 (14) Å	µ = 0.49 mm^−^^1^
*c* = 12.9784 (12) Å	*T* = 113 K
β = 99.120 (7)°	Prism, colorless
*V* = 998.44 (18) Å^3^	0.20 × 0.18 × 0.10 mm
*Z* = 2	

### Data collection

**Table:**

Rigaku Saturn CCD area-detector diffractometer	2384 independent reflections
Radiation source: rotating anode	1870 reflections with *I* > 2σ(*I*)
multilayer	*R*_int_ = 0.038
Detector resolution: 14.63 pixels mm^-1^	θ_max_ = 27.9°, θ_min_ = 2.2°
φ and ω scans	*h* = −7→7
Absorption correction: multi-scan (*CrystalClear*; Rigaku/MSC, 2005)	*k* = −17→17
*T*_min_ = 0.908, *T*_max_ = 0.953	*l* = −15→16
9992 measured reflections	

### Refinement

**Table:**

Refinement on *F*^2^	Primary atom site location: structure-invariant direct methods
Least-squares matrix: full	Secondary atom site location: difference Fourier map
*R*[*F*^2^ > 2σ(*F*^2^)] = 0.031	Hydrogen site location: inferred from neighbouring sites
*wR*(*F*^2^) = 0.088	H-atom parameters constrained
*S* = 1.06	*w* = 1/[σ^2^(*F*_o_^2^) + (0.0495*P*)^2^] where *P* = (*F*_o_^2^ + 2*F*_c_^2^)/3
2384 reflections	(Δ/σ)_max_ = 0.001
127 parameters	Δρ_max_ = 0.49 e Å^−^^3^
0 restraints	Δρ_min_ = −0.22 e Å^−^^3^

### Special details

**Table:**

Geometry. All e.s.d.'s (except the e.s.d. in the dihedral angle between two l.s. planes) are estimated using the full covariance matrix. The cell e.s.d.'s are taken into account individually in the estimation of e.s.d.'s in distances, angles and torsion angles; correlations between e.s.d.'s in cell parameters are only used when they are defined by crystal symmetry. An approximate (isotropic) treatment of cell e.s.d.'s is used for estimating e.s.d.'s involving l.s. planes.
Refinement. Refinement of *F*^2^ against ALL reflections. The weighted *R*-factor *wR* and goodness of fit *S* are based on *F*^2^, conventional *R*-factors *R* are based on *F*, with *F* set to zero for negative *F*^2^. The threshold expression of *F*^2^ > σ(*F*^2^) is used only for calculating *R*-factors(gt) *etc*. and is not relevant to the choice of reflections for refinement. *R*-factors based on *F*^2^ are statistically about twice as large as those based on *F*, and *R*- factors based on ALL data will be even larger.

### Fractional atomic coordinates and isotropic or equivalent isotropic displacement parameters (Å^2^)

**Table:**

	*x*	*y*	*z*	*U*_iso_*/*U*_eq_	
S1	1.18924 (6)	0.48167 (3)	0.35236 (3)	0.01929 (12)	
S2	0.94277 (7)	0.60479 (3)	0.17655 (3)	0.02206 (13)	
N1	0.8027 (2)	0.39565 (9)	0.36814 (10)	0.0221 (3)	
N2	0.7508 (2)	0.46308 (10)	0.28684 (10)	0.0206 (3)	
C1	1.3577 (3)	0.34573 (11)	0.54385 (12)	0.0210 (3)	
H1	1.4506	0.3955	0.5185	0.025*	
C2	1.4532 (3)	0.28628 (12)	0.62747 (12)	0.0213 (3)	
H2	1.6099	0.2969	0.6601	0.026*	
C3	1.3224 (3)	0.21206 (12)	0.66341 (12)	0.0237 (4)	
H3	1.3889	0.1710	0.7199	0.028*	
C4	1.0921 (3)	0.19787 (12)	0.61620 (13)	0.0259 (4)	
H4	1.0013	0.1469	0.6409	0.031*	
C5	0.9936 (3)	0.25738 (12)	0.53341 (12)	0.0216 (3)	
H5	0.8363	0.2469	0.5015	0.026*	
C6	1.1262 (3)	0.33270 (11)	0.49711 (11)	0.0173 (3)	
C7	1.0225 (3)	0.39663 (11)	0.40992 (12)	0.0171 (3)	
C8	0.9342 (3)	0.51325 (11)	0.27088 (12)	0.0175 (3)	
C9	0.6507 (3)	0.59299 (11)	0.10328 (12)	0.0211 (3)	
H9A	0.5373	0.5895	0.1527	0.025*	
H9B	0.6143	0.6531	0.0597	0.025*	
C10	0.6215 (2)	0.50122 (11)	0.03322 (12)	0.0205 (3)	
H10A	0.6445	0.4405	0.0769	0.025*	
H10B	0.7419	0.5018	−0.0130	0.025*	

### Atomic displacement parameters (Å^2^)

**Table:**

	*U*^11^	*U*^22^	*U*^33^	*U*^12^	*U*^13^	*U*^23^
S1	0.01581 (19)	0.0215 (2)	0.0199 (2)	−0.00349 (15)	0.00081 (16)	0.00081 (15)
S2	0.0213 (2)	0.0229 (2)	0.0209 (2)	−0.00350 (16)	−0.00005 (17)	0.00217 (16)
N1	0.0185 (7)	0.0276 (7)	0.0199 (7)	−0.0024 (6)	0.0025 (6)	0.0024 (6)
N2	0.0171 (6)	0.0260 (7)	0.0185 (7)	−0.0008 (6)	0.0021 (5)	0.0017 (5)
C1	0.0198 (8)	0.0196 (8)	0.0232 (9)	−0.0048 (6)	0.0025 (7)	−0.0012 (6)
C2	0.0160 (7)	0.0247 (8)	0.0224 (9)	−0.0007 (6)	−0.0003 (6)	−0.0022 (7)
C3	0.0249 (8)	0.0251 (9)	0.0208 (8)	0.0021 (7)	0.0027 (7)	0.0039 (7)
C4	0.0235 (8)	0.0267 (9)	0.0286 (9)	−0.0040 (7)	0.0072 (7)	0.0057 (7)
C5	0.0155 (7)	0.0267 (8)	0.0223 (9)	−0.0040 (6)	0.0025 (6)	−0.0008 (7)
C6	0.0189 (7)	0.0175 (7)	0.0159 (8)	−0.0003 (6)	0.0041 (6)	−0.0036 (6)
C7	0.0168 (7)	0.0184 (7)	0.0169 (8)	−0.0024 (6)	0.0052 (6)	−0.0038 (6)
C8	0.0172 (7)	0.0208 (8)	0.0140 (8)	0.0004 (6)	0.0010 (6)	−0.0040 (6)
C9	0.0194 (8)	0.0221 (8)	0.0207 (9)	0.0017 (6)	−0.0008 (7)	0.0015 (6)
C10	0.0184 (8)	0.0224 (8)	0.0194 (8)	0.0023 (6)	−0.0007 (6)	0.0003 (6)

### Geometric parameters (Å, °)

**Table:**

S1—C8	1.7289 (15)	C3—H3	0.9500
S1—C7	1.7400 (15)	C4—C5	1.388 (2)
S2—C8	1.7417 (16)	C4—H4	0.9500
S2—C9	1.8126 (15)	C5—C6	1.397 (2)
N1—C7	1.303 (2)	C5—H5	0.9500
N1—N2	1.3872 (17)	C6—C7	1.471 (2)
N2—C8	1.303 (2)	C9—C10	1.526 (2)
C1—C2	1.390 (2)	C9—H9A	0.9900
C1—C6	1.393 (2)	C9—H9B	0.9900
C1—H1	0.9500	C10—C10^i^	1.531 (3)
C2—C3	1.379 (2)	C10—H10A	0.9900
C2—H2	0.9500	C10—H10B	0.9900
C3—C4	1.391 (2)		
			
C8—S1—C7	86.82 (7)	C1—C6—C7	120.63 (14)
C8—S2—C9	100.29 (7)	C5—C6—C7	120.18 (13)
C7—N1—N2	112.95 (13)	N1—C7—C6	124.58 (14)
C8—N2—N1	112.01 (12)	N1—C7—S1	113.64 (12)
C2—C1—C6	120.32 (15)	C6—C7—S1	121.78 (11)
C2—C1—H1	119.8	N2—C8—S1	114.58 (12)
C6—C1—H1	119.8	N2—C8—S2	126.30 (12)
C3—C2—C1	120.49 (14)	S1—C8—S2	119.11 (9)
C3—C2—H2	119.8	C10—C9—S2	112.94 (11)
C1—C2—H2	119.8	C10—C9—H9A	109.0
C2—C3—C4	119.44 (15)	S2—C9—H9A	109.0
C2—C3—H3	120.3	C10—C9—H9B	109.0
C4—C3—H3	120.3	S2—C9—H9B	109.0
C5—C4—C3	120.70 (15)	H9A—C9—H9B	107.8
C5—C4—H4	119.6	C9—C10—C10^i^	111.00 (16)
C3—C4—H4	119.6	C9—C10—H10A	109.4
C4—C5—C6	119.84 (15)	C10^i^—C10—H10A	109.4
C4—C5—H5	120.1	C9—C10—H10B	109.4
C6—C5—H5	120.1	C10^i^—C10—H10B	109.4
C1—C6—C5	119.19 (14)	H10A—C10—H10B	108.0
			
C7—N1—N2—C8	−0.54 (19)	C1—C6—C7—S1	6.9 (2)
C6—C1—C2—C3	1.7 (2)	C5—C6—C7—S1	−172.53 (12)
C1—C2—C3—C4	−0.9 (2)	C8—S1—C7—N1	0.48 (12)
C2—C3—C4—C5	0.2 (3)	C8—S1—C7—C6	−179.80 (13)
C3—C4—C5—C6	−0.2 (3)	N1—N2—C8—S1	0.93 (17)
C2—C1—C6—C5	−1.8 (2)	N1—N2—C8—S2	−179.93 (11)
C2—C1—C6—C7	178.80 (14)	C7—S1—C8—N2	−0.81 (13)
C4—C5—C6—C1	1.1 (2)	C7—S1—C8—S2	179.98 (10)
C4—C5—C6—C7	−179.52 (15)	C9—S2—C8—N2	−7.85 (16)
N2—N1—C7—C6	−179.79 (13)	C9—S2—C8—S1	171.26 (9)
N2—N1—C7—S1	−0.08 (17)	C8—S2—C9—C10	−75.00 (13)
C1—C6—C7—N1	−173.44 (15)	S2—C9—C10—C10^i^	−175.61 (14)
C5—C6—C7—N1	7.2 (2)		

Symmetry codes: (i) −*x*+1, −*y*+1, −*z*.

### Hydrogen-bond geometry (Å, °)

**Table:**

Cg is the centroid of the C1–C6 ring.

**Table:**

*D*—H···*A*	*D*—H	H···*A*	*D*···*A*	*D*—H···*A*
C9—H9B···Cg^ii^	0.99	2.70	3.540 (2)	144

Symmetry codes: (ii) −*x*+1, *y*+1/2, −*z*+1/2.
